# Extreme tolerance for nocturnal emergence at low body temperatures in a high-latitude lizard: implications for future climate warming

**DOI:** 10.1093/conphys/coac082

**Published:** 2023-01-21

**Authors:** Christian O Chukwuka, Joanne M Monks, Alison Cree

**Affiliations:** Department of Zoology, University of Otago, Dunedin 9016, Aotearoa New Zealand; Department of Biology, Alex Ekwueme Federal University, Ndufu-Alike Ikwo 482131, Ebonyi State, Nigeria; Department of Zoology, University of Otago, Dunedin 9016, Aotearoa New Zealand; Biodiversity Group, Department of Conservation, Dunedin 9058, Aotearoa New Zealand; Department of Zoology, University of Otago, Dunedin 9016, Aotearoa New Zealand

**Keywords:** Active body temperature, cold adaptation, nocturnal emergence, nocturnality, operative temperature, thermography, trail camera, winter activity

## Abstract

High-latitude lizards live in environments where ambient air temperature at night is frequently below retreat temperatures, which likely has implications for nocturnal emergence and activity. However, patterns of lizard activity at night under current temperate climates are poorly understood, a situation that limits our understanding of potential effects of climate change. We investigated patterns of nocturnal emergence and activity in the cold-adapted, viviparous gecko (*Woodworthia* ‘Otago/Southland’). We measured operative environmental temperature (*T*_e_) available to geckos that emerged at night and simultaneously assessed nighttime emergence activity using time-lapse trail cameras. Also, we assessed field body temperature (*T*_b_) of emerged geckos of various life history groups at night using thermography to understand how current weather conditions affect field *T*_b_ of emerged geckos. Our results show that *T_e_*, nocturnal emergence activity and field-active *T*_b_ increased with nighttime air temperature. Nocturnal emergence was highest in spring and summer but also occurred in autumn and (unexpectedly) in winter. Geckos were active over a broad range of *T*_b_ down to 1.4°C (a new record low for lizards) and on rock surfaces typically warmer than air temperature or *T*_b_. We conclude that this nocturnal, high-latitude lizard from the temperate zone is capable of activity at low winter temperatures, but that current climate limits emergence and activity at least in autumn and winter. Activity levels for cool-temperate reptiles will probably increase initially as climates warm, but the consequences of increased nocturnal activity under climate change will probably depend on how climate change affects predator populations as well as the focal species’ biology.

## Teaser

High-latitude lizards are capable of activity at low winter temperatures and are active on warm nights (including in winter) with prior warmer temperatures during the day, with active field body temperature as low as 1.4#x00B0;C and air temperature less than 1#x00B0;C.

## Introduction

Activity in ectotherms is primarily dependent on their immediate thermal environment (Sound and Veith, 2000; Angilletta, 2009). Ectotherms make the best use of their immediate environment when weather variables such as air temperature, solar radiation, cloud cover, wind and photoperiod are favourable for activity (Gordon *et al.,* 2010; Vermunt *et al.,* 2014). Extremes of these variables result in a significant decline in fitness (Tewksbury *et al.,* 2008), and a persistent change in weather variables can be lethal, for example leading to cold death (Stroud *et al.,* 2020) or local extinction (Huey and Tewksbury, 2009; Sinervo *et al.,* 2010). In extreme cold at near-freezing temperatures, metabolic rate of reptiles falls to low levels, contraction of isolated muscles declines and locomotory ability eventually ceases (Paladino, 1985; Storey, 2006; James, 2013).

Although temperature plays a fundamental role in determining when squamates are active, including when thermoregulating, foraging or even mating (Adolph and Porter, 1993; Autumn *et al.,* 1994; Logan *et al.,* 2015; Gunderson and Leal, 2016), other factors, including wind, solar radiation and water availability, are also influential. For example, wind can counter the effect of warm temperatures on lizards through convective cooling of the skin surfaces; it can force squamates to remain in a retreat by decreasing air and substrate temperature and also by affecting water relations (Kearney and Porter, 2009; Ortega *et al.,* 2017). Thus, wind can reduce activity time, thermoregulatory efficiency and thermal quality (Logan *et al.,* 2015; Ortega *et al.,* 2017). Water availability is also a strong predictor of activity in squamates and high rates of evaporative water loss can restrict activity, which helps avoid further water loss. Lizards’ activity increased after a heavy rainfall event (Kearney *et al.,* 2018) and low water availability such as a dry spell can result in reduced activity with animals remaining in retreats (Crowley, 1987; Lorenzon *et al.,* 1999; Kearney *et al.,* 2018). Vapour pressure deficit (VPD) facilitates water loss through the integument due to its drying power, forcing an animal to remain in its retreat (Kearney *et al.,* 2018). However, the effect of VPD may be less noticeable at night and not predict activity patterns of nocturnal squamates due to the lower temperatures.

Furthermore, in some species, activity is not only dependent on weather variables, but also on ecological needs and functions (Brown and Shine, 2002) and on life history traits such as differences between juveniles and adults (Adolph and Porter, 1993). Behavioural plasticity can also help shape lizard activity, even when weather conditions are favourable (López-Alcaide *et al.,* 2017). Regardless of how they are influenced, activity periods determine how the ecological niches of lizards are shaped, including how lizards interact with competitors for mates and food resources and, potentially, their exposure to predators (Anholt *et al.,* 2000; López-Alcaide *et al.,* 2017). However, for higher latitude cool-climate lizards, how weather conditions shape activity, the influence of life history group (which can include body size differences) on these patterns and the extent to which cold winter weather forces these species to remain in retreats remain unclear.

Nocturnality poses particular constraints on activity of some high-latitude ectotherms. In general, nocturnality signals an ability to be active at relatively low nighttime environmental temperatures (Hare *et al.,* 2005) and at low body temperature (Autumn *et al.,* 1994); it brings advantages in reducing dietary competition with sympatric, diurnal organisms (Vitt *et al.,* 2003) and in reducing exposure to primarily diurnal, visually oriented predators (Gaston, 2019), while still allowing high body temperature (*T_b_*) to be achieved during the day (Huey *et al.,* 1989). Despite experiencing relatively low and variable temperatures for activity at night, when *T_b_* typically falls below preferred body temperature (Huey *et al.,* 1989; Autumn *et al.,* 1997), nocturnal lizards can still be capable of high locomotory function (Autumn *et al.,* 1997). High levels of nocturnal activity are apparent in some ectotherms where air temperatures are high (Weatherhead *et al.,* 2012; Sperry *et al.,* 2013), but even at higher latitudes with low (~8°C) air temperature at dusk, field activity in cold-adapted species can be high at night (Hare *et al.,* 2016). Nonetheless, a cessation of activity throughout winter at high latitudes is often observed with high-latitude lizards remaining in retreats during normal activity time.

Thus, increased nighttime temperature could be beneficial to cool-temperate lizards by creating a thermal environment that enhances activity. Increased nighttime temperature is particularly driven by increasing cloudiness during the day, which helps retain heat on the surface (Cox *et al.,* 2020). As climate change raises temperatures (including nocturnal temperatures) and affects other weather conditions around the globe (IPCC, 2021), it becomes important to understand the ways that current and future conditions might affect activity of ectotherms, including nocturnal, high-latitude species (Deutsch *et al.,* 2008; Johansson *et al.,* 2020).

Here, we investigated the nocturnal activity pattern of a cold-adapted, viviparous gecko known by the tag name *Woodworthia* ‘Otago/Southland’ (Nielsen *et al.,* 2011; Hitchmough *et al.,* 2021), at a subalpine site (Macraes) in southern Aotearoa New Zealand, to understand how current weather conditions influence nocturnal emergence. This rock-dwelling taxon is recognized as a nocturnal forager (Whitaker, 1984; Spencer and Grimmond, 1994), though it has an unusual activity pattern at our study site in being active outside the retreat not only at nighttime but also when basking cryptically during the day at the retreat entrance (Gibson *et al.,* 2015); such basking, seen almost entirely in pregnant females (Chukwuka, 2020), helps elevate *T_b_* both in the field and the laboratory and thereby hastens embryonic development (Cree and Hare, 2016a). Ambient air temperature at Macraes is frequently below the geckos’ preferred body temperature (Rock *et al.,* 2000) and also below retreat-site temperature (Chukwuka *et al.,* 2021). Although it is known that air temperature during nocturnal activity can be less than 8°C (Chukwuka *et al.,* 2019), the way that current weather conditions shape nighttime emergence activity and field body temperature, including whether these patterns differ intraspecifically, remains unclear.

We assessed the seasonal variation in operative environmental temperature (*T*_e_) available to geckos when they emerged in the open at night using dataloggers inserted into lizard models. Concurrently, we assessed nighttime emergence activity using time-lapse trail cameras (thus eliminating any effect of observer presence). We explored whether weather conditions influence field *T*_e_ and nocturnal emergence and whether the effects vary with season, time after dusk, retreat type and life history group (adult versus juvenile, male versus female). We predicted that activity in these geckos would be high in spring and summer when the air temperature is high compared to winter and that geckos would be inactive in winter due to low temperatures. We also predicted that geckos’ emergence from thin rock slabs would be less than from thick rock slabs and deep crevices. Thin rock slabs cool rapidly at dusk and geckos may select retreats with more stable temperatures at night (Chukwuka *et al.,* 2021). Nighttime emergence was also predicted to be higher on nights with prior warmer daytime temperatures than following cold days. We also quantified the field body temperature (*T*_b_) of emerged geckos of different life history groups at night, using a thermal infrared camera, to understand how weather conditions and nighttime rock surface temperature affect field *T*_b_. For emerged geckos, we predicted that night field *T*_b_ would be higher than the *T*_a_ and closer to rock surface temperature, noting that rock surfaces can remain warmer than air temperature following warm days and this warmth would be beneficial to gecko activity. In addition, we predicted that larger geckos (adults) and pregnant female geckos would have higher *T*_b_ that is more independent of *T*_a_ (more similar to retreat-site temperature) in the first few hours after dusk than small geckos (juveniles) and other life history groups, respectively, and that these trends would be seasonally dependent. We expected these differences in *T*_b_ given the effects of body size on rate of heat loss and given the higher thermal preference of geckos during pregnancy, which hastens embryonic development (Rock *et al.,* 2000; Cree and Hare, 2016b). Our study provides a detailed insight into activity patterns under the current climate and insight into future possible impacts of climate change.

## Materials and methods

### Study site

Our study was conducted near Macraes township (−45° S, 550–710 m asl), eastern Otago, Aotearoa New Zealand, between May 2017 and April 2019. Field sampling was conducted in two adjacent locations: The Department of Conservation’s Redbank Reserve for surveys of nighttime field body temperature and nearby private land for filming, both within 4 km of one another. The rock tors in these field sites have loose slabs and horizontal cracks (deep crevices) inhabited by geckos (Rock *et al.,* 2002), and surrounding the tors at the base are tussock grasses and some fruiting shrubs, including *Chionochloa rubra, C. rigida* and *Festuca novae-zelandiae*.

### Measurement of nighttime operative environmental temperature

We deployed six hollow copper models calibrated against gecko body temperatures (Penniket and Cree, 2015) to measure operative environmental temperature (*T_e_*) available to emerged geckos at night (Bakken, 1992). Thermocron iButtons (DS1921G-F5#, resolution ±0.5°C from −30°C to +70°C, recording hourly) were inserted into each copper model, and the model was sealed with ultraviolet-resistant tape (Rock *et al.,* 2000; Dzialowski, 2005). Each copper model was glued to a terracotta tile and positioned on a rock tor close to a retreat-site entrance. *T_e_* was measured concurrent with the activity filming and is presented for the first 5 hours after dusk; this was the period when nocturnal activity was at its peak (Christian Chukwuka, *pers. obs.*).

### Patterns of nocturnal emergence of geckos using trail cameras

We conducted field surveys for 28 days in each season using nine trail cameras (Reconyx™ wildlife cameras) in three types of retreats: thin loose slabs (thickness: ≤4.5 cm), thick loose slabs (thickness: ≥4.6 cm) and horizontal crevices of >0.4 m deep within rock outcrops (Chukwuka *et al.,* 2021). At the start of the first season of filming in spring, turnable rock slabs (thick and thin loose slabs) were turned to confirm that at least one or more geckos from each life history group (adult male and female, snout–vent length (SVL) ≥ 68 mm; and juvenile, SVL < 68 mm) were present (Rock *et al.,* 2000). Geckos inside deep crevices were not able to be captured or marked. The geckos captured under the turnable rock slabs were marked with a non-toxic silver pen in the spring season only, on both flanks, for easy identification in the photo frame. The geckos were returned to the capture site immediately after handling.

The cameras were mounted on metal stakes with a ball-head camera mount and positioned level with the retreat site at a 1-m distance from the rock tor (one camera per crevice or slab); they were then set using a time-lapse function to capture photos of the retreats and their surroundings (Hobbs and Brehme, 2017), using infrared night vision. We used a time-lapse function rather than motion sensors due to the geckos’ small body size and insufficient temperature difference from background temperature (Welbourne *et al.,* 2017). The images were processed using online free software, Timelapse2, which collates data directly to an Excel sheet (Greenberg, 2017). We played a series of photo frames to detect geckos by looking for eye shine and movement of geckos among frames. The camera footage from dusk until 5 h after dusk was examined to assess the geckos’ nocturnal activity; nocturnal emergence was taken as the appearance of half or more of the gecko’s body outside the retreat. The number of geckos seen emerged in each frame and emergence duration, inferred from the presence of a gecko in the same position on images at 1-min intervals, were quantified. Due to the inability to differentiate between adult sexes and reproductive conditions of the geckos from the monochrome photos, we categorized the life history group only as an adult or juvenile for this aspect of the study.

All the footage was examined twice by one person for the presence or absence of geckos in each image. We assumed that likelihood of detecting geckos was constant for all the photo frames. There was no data loss or camera malfunction for the nine cameras installed, except when one camera was attacked by a non-native brushtail possum, *Trichosurus vulpecula*, during the spring season. As this disturbance changed the field of view for 10 recording days from the targeted rock slab, these recordings were excluded from the analysis.

### Field body temperature and rock-tor surface temperature at night

At an adjacent site, we sampled emerged geckos at night to measure field body temperatures in each season. Using spotlights for the 5 h after dusk, we detected geckos from a distance by eye-shine or from their bodies at closer approach (Lettink and Monks, 2016). We measured skin surface temperature (dorsal abdomen) using a thermal infrared camera (IRC, Flir i60) or with a mini-infrared thermometer without touching or disturbing the gecko (Chukwuka *et al.,* 2019). The two devices were used interchangeably because the IRC battery depleted before the night survey started on some days. The data measured with the two devices were standardized using a calibration equation in Chukwuka *et al*. (2019). We captured the geckos to measure their SVL to differentiate adults and juveniles and also determined the females’ reproductive status (Rock *et al.,* 2000; Cree and Hare, 2016b). We distinguished sex of adults by the presence of a hemipenial sac at the base of the tail as well as pre-anal and femoral pores anterior to the cloacal opening in males. We distinguished female reproductive condition (pregnant vs non-pregnant) by palpation. We released the geckos at the same site within 1 min of capture.

In addition, we also measured the rock-tor surface temperatures surrounding where the geckos were captured to infer substrate temperature at night. A circular region of interest (ROI) was drawn around the geckos and the maximum temperature within the ROI was taken as the substrate temperature where geckos were captured.

### Field retreat temperatures

Daytime field retreat temperatures (*T*_retreat_) were measured using Thermocron iButtons to test whether it predicts emergence at night. Six Thermocron iButtons were installed in each of three different retreat types, i.e. thick and thin rock slabs and deep crevices (Chukwuka *et al.,* 2020; Chukwuka *et al.,* 2021), using duct tape. For the deep crevices, iButtons were inserted ~0.4 m depth with insulated metal wires. We set the iButtons to measure the temperature every hour and analysed data from the first five hours after dusk to be consistent with filming.

### Climate data

We obtained weather conditions (air temperature, rainfall, relative humidity and wind speed) recorded every 60 min during the study period at the weather station (Fire and Emergency New Zealand, FENZ) located ~ 2 km from the study sites from National Institute of Water and Atmospheric Research. Solar radiation data measured hourly using a pyranometer installed beside the FENZ weather station were also obtained (Evie Virens, University of Otago, pers. comm.).

### Data analysis

All data were analysed in R package (R version 3.5.3), and plots were generated with the ‘ggplot2’ package in using Rstudio interface (R Core Team, 2008). Data were presented as mean ± standard error with the confidence limit set at 95%. We tested for homogeneity of the dataset using the plot of residuals in ‘autoplot’ function in R. All skewed data were log-transformed before the final analysis. For significant results, effect sizes (Hedges *g*) were calculated to compare mean differences and interpreted as very small (0.01–0.19), small (0.20–0.49), medium (0.50–0.79), large (0.80–1.20) and very large (>1.20) (Sawilowsky, 2009).

### Field operative temperature

We analysed the hourly mean field *T_e_* using multiple linear regression to determine the influence of current weather conditions (Shine and Kearney, 2001). Mean differences in *T_e_* across seasons and times of day were compared using a two-way ANOVA. We calculated the drying power of the air, i.e. VPD, using temperature and relative humidity (Jucker *et al.,* 2018). We presented *T_e_* relative to a measure of preferred body temperature calculated for the same population of geckos from the raw data of a published study (Rock *et al.,* 2000), to infer if the activity temperature matches the gecko’s preferred body temperature. We calculated the 50th percentile of the preferred body temperature selected by the geckos at 21:30 h in spring and summer for adult geckos.

### Nighttime emergence in relation to season, time of night and weather conditions

To understand the influence of weather conditions (air temperature, wind speed, VPD and rainfall) on counts of gecko sightings per hour within 5 h after dusk, we modelled camera data with a generalized linear mixed model using template model builder as implemented in the R package glmmTMB (version 1.1.5) using the functions, glmmTMB and a nbinom2 family (Booth *et al.,* 2003; Brooks *et al.,* 2017). The ‘glmmTMB’ package estimates zero inflation in a dataset and models random effects following repeated sampling using Laplace approximation (Brooks *et al.,* 2019). The excess zeros in the dataset were regarded as a true zero because the frequency of gecko activity depends on real ecological effects such as suitable weather variables for activity and the presence/absence of predators around the retreats (Welsh *et al.,* 1996; Martin *et al.,* 2005). For this model, season and retreat type were included as fixed factors. We checked for multicollinearity using variance inflation factor score, and *T_e_* was removed from the final model due to collinearity with *T_a_*. We retained *T_a_* rather than *T*_e_ in the model as it is from the same weather station/location and points in time as the other weather variables used in the analysis. Also, the effects of the season, retreat type and life history stage (adult or juvenile) on the duration of nocturnal emergence were modelled using a separate glmmTMB model. For all these models, camera identity was included as a random factor. We included interaction terms for variables that have biological significance.

Also, we tested how warmer daytime temperature (daytime *T*_a_) influenced the nocturnal emergence of the geckos using a generalized linear model with counts of gecko emergence at night as the dependent variable and mean daytime air temperature as a predictor variable. We included seasons in the model as a fixed co-factor. We also calculated the difference between the daytime mean air temperature and both nighttime *T*_retreat_ and *T*_e_ to test whether these variables affected the number of lizards that emerged from the retreat at night.

### Active field body temperature

To understand how season and life history group influenced nighttime field body temperature, we performed a two-way ANOVA using mean field *T_b_* as a response variable. For the body temperature analysis only, the time when the geckos were captured was included as a covariate. We also performed a linear regression to establish relationships between the gecko’s field body temperature and nighttime air temperature and between *T_b_* and substrate temperature where the geckos were captured to identify the best predictor of field *T_b_*. Also, the influence of weather conditions on nighttime field body temperature was modelled simultaneously using multiple linear regression, with the season and hours after dusk as fixed factors.

## Results

### Field operative temperatures

**Figure 1:**
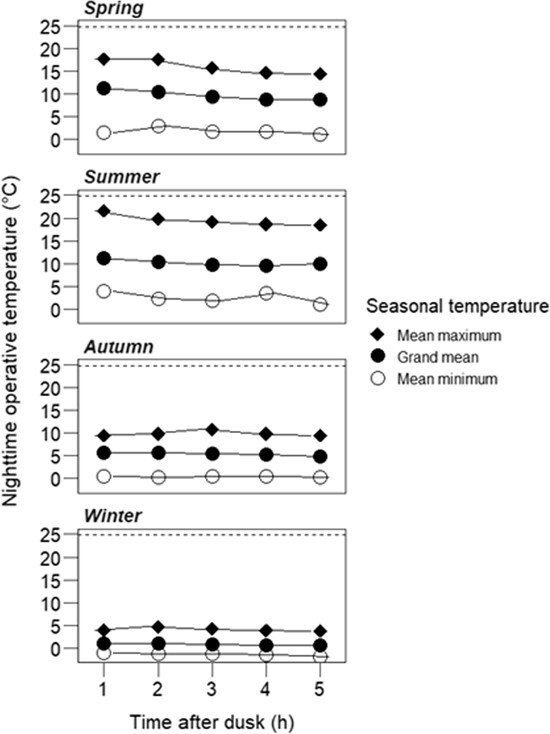
Hourly mean operative temperatures (*T_e_*) on the exposed rock surface during 28 days of each season at Macraes, Otago, New Zealand. Mean *T_e_* differed significantly across the seasons and with time after dusk (*P* < 0.001). There was also a significant interaction (*P* < 0.001), with the effect of time after dusk being greatest in spring and summer. Data were collated only for the first 5 hours after dusk when the activity of geckos was high in the field. *N* = 6 copper models. Standard errors are small and within the size of the plot shapes. The dotted line represents the 50th percentile of preferred body temperature selected by the same population of geckos at 21:30 hr in spring and summer (Rock *et al.,* 2000).

The nighttime operative environmental temperature (*T_e_*) during the filming periods reached highest values in summer (mean maximum, 21.7°C) and lowest in winter (mean minimum −1.92°C) and differed significantly in autumn and winter compared to spring and summer (χ^2^ = 557.06, *df* = 3, *P* < 0.001; Figure 1). The mean maxima of the *T*_e_ were always below the geckos’ preferred body temperature (25°C) from a published study (Rock *et al.,* 2000) in all the seasons. *T_e_* showed a decline from the first hour after dusk until the fifth hour (χ^2^ = 18.02, *df* = 1, *P* < 0.001), the decline being greater in spring and summer than in autumn and winter (season^*^time after dusk: χ^2^ = 5.83, *df* = 12, *P* < 0.001).

All the weather conditions influenced the field *T_e_* (*P < 0.001*, Table 1), with a positive relationship between *T_e_* and air temperature (*r* = 0.69) and a negative relationship between *T_e_* and wind speed (*r* = −0.23, Supplementary information Figure S1). The relationship between *T*_a_ and *T*_e_ was affected significantly by wind speed, VPD and amount of rainfall (*P* < 0.001, Table 1).

**Table 1:** Summary statistics of multiple regression between weather variables and the mean operative temperature (*T*_e_) of copper models.

**Weather variable**	***F***	***df***	***P***
Air temperature (°C)	1645	1	**<0.001**
Wind speed (m/s)	49.42	1	**<0.001**
VPD (kPa)	282.84	1	**<0.001**
Rainfall (mm)	28.75	1	**<0.001**
Air temperature^*^wind speed	10.71	1	**<0.001**
Air temperature^*^VPD	40.45	1	**<0.001**

Copper models were placed in potential positions for basking and nocturnal emergence in the habitat of the gecko *Woodworthia* ‘Otago/Southland’ at Macraes, Otago, New Zealand from May 2017 to April 2019. Data for all the weather variables except solar radiation were sourced from the Fire and Emergency New Zealand weather station at Macraes; data for solar radiation were provided by Evie Virens (University of Otago, pers. comm.). Significant results are indicated in boldface.

### Nighttime emergence in relation to season, time of night and weather conditions

**Figure 2:**
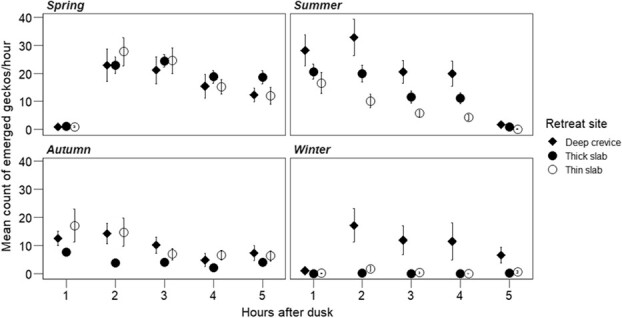
Hourly mean number of gecko sightings across the hour (from 60 images per trail camera) for 28 days in each season, for *Woodworthia ‘*Otago/Southland’ at Macraes, Otago, New Zealand. Nocturnal emergence differed significantly among the retreat types and seasons (*P* < 0.05) but not with time after dusk (*P* > 0.05, *N* = 2–4 cameras for each retreat type).

Activity filming using trail cameras yielded 51,153 sightings of geckos from the photographs examined from the first 5 h after dusk. Most geckos sighted at night were adults (88%) rather than juveniles (12%). The number of gecko sightings in every 60 images (1 h) of footage varied significantly with season (χ^2^ = 22.27, *df* = 3, *P* = < 0.001) and with time after dusk (χ^2^ = 172.80, *df* = 4, *P* < 0.001; Figure 2, Table 2). Emergence was relatively high in spring and summer (means up to 33 sightings per hour in spring) but reduced in autumn and winter (means up to 17 sightings per hour; Figure 2). No significant effect of retreat type (thick rock slab, thin rock slab or deep crevice) on mean number of geckos sighted was observed (χ^2^ = 1.36, *df* = 2, *P* = 0.51). However, there were more sightings of geckos that emerged from deep crevices in summer and winter than for other retreat types (interaction of season with retreat type: χ^2^ = 33.19, *df* = 6, *P* < 0.001). A larger number of geckos were sighted in the first 2 hours after dusk in summer than at other times (interaction of season with time after dusk: χ^2^ = 706.27, *df* = 12, *P* < 0.001).

**Table 2:** Summary statistics of the ‘glmmTMB’ model for nocturnal emergence of the gecko *Woodworthia* ‘Otago/Southland’ at Macraes, Otago, New Zealand

**Predictor**	***χ*** ^***2***^	***df***	***P***
Time after dusk	172.79	4	**<0.001**
Season	22.27	3	**<0.001**
Retreat type	1.36	2	0.52
Season: time after dusk	79.20	3	**<0.001**
Season: retreat type	38.16	6	**<0.001**
**Weather variable**			
Air temperature (°C)	56.71	1	**<0.001**
Wind speed (m/s)	31.41	1	**<0.001**
VPD (kPa)	1.98	1	0.16
Rainfall (mm)	0.0004	1	0.98
Air temperature: wind speed	27.28	1	**<0.01**
Air temperature: VPD	4.57	1	0.03

The response variable was the number of sightings in 60 frames taken at 1-min intervals with nine time-lapse trail cameras. The significant variables are indicated in boldface.

Nocturnal emergence increased with increasing air temperature at the time of emergence (χ^2^ = 56.71, *df* = 1, *P* < 0.001) and with decreasing wind speed (between 0 and 5 m/s; χ^2^ = 31.41, *df* = 1, *P* < 0.001; Table 2, Figure S2). VPD and rainfall did not predict gecko emergence at night (*P* > 0.05 for both; Table 2, Figure S2). The effect of air temperature on nocturnal emergence also depended on the combined influence of air temperature and wind speed (air temperature: wind speed, χ^2^ = 27.28, *df* = 1, *P* < 0.01).

**Figure 3:**
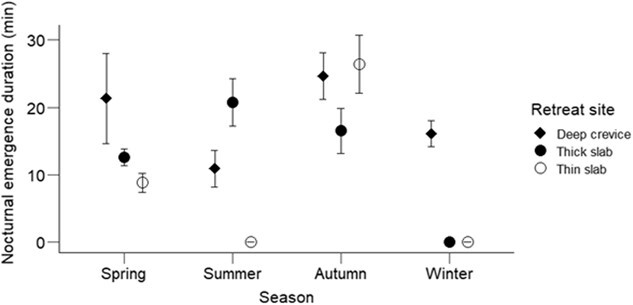
Mean ± SE duration per bout spent outside the retreat by *Woodworthia* ‘Otago/Southland’ geckos during nighttime emergence at Macraes, Otago, New Zealand. In winter, emerged geckos were sighted only from deep crevices. In summer, no emerged gecko was sighted from under thin rock slabs. The nocturnal emergence duration differed significantly across seasons (*P* = 0.001) but not among retreat types (*P* = 0.62). However, the interaction of season and retreat type was significant (*P* = 0.02). The emergence duration was inferred from the presence of a gecko in the same position on images per trail camera taken at 1-min intervals.

The emergence duration, an inferred bout of emergence for individuals seen over several photo frames, differed significantly across the seasons (χ^2^ = 12.51, *df* = 3, *P* = 0.001) but not with retreat type (χ^2^ = 0.95, *df* = 2, *P* = 0.62; Figure 3). Emergence duration was longer in spring at the entrance to the deep crevice compared to other seasons and retreat types (season: retreat type, χ^2^ = 9.23, *df* = 3, *P* = 0.02). However, adult (16.2 ± 1.07 min) and juvenile geckos (22 ± 3.5 min) spent similar amounts of time emerged per bout at night (χ^2^ = 0.87, *df* = 1, *P* = 0.35) with small effect size (Hedges *g* = 0.28).

Nocturnal emergence is more likely to be high on nights with prior warmer temperature during the day (χ^2^ = 7.24, *df* = 1, *P* = 0.007), and with more observed effect in winter and autumn than in spring and summer (χ^2^ = 15.01, *df* = 3, *P* = 0.001; Supplementary information Figure S3) as confirmed by a significant interaction (daytime temperature*season: χ^2^ = 28.22, *df* = 3, *P* < 0.001). However, nocturnal emergence activity was not affected by the difference between retreat temperature at night and daytime heating air temperature (χ^2^ = 0.43, *df* = 1, *P* = 0.51) nor *T*_e_ (χ^2^ = 2.45, *df* = 1, *P* = 0.11); Supplementary information Figure S4).

### Nighttime field active body temperature and rock surface temperature

**Figure 4:**
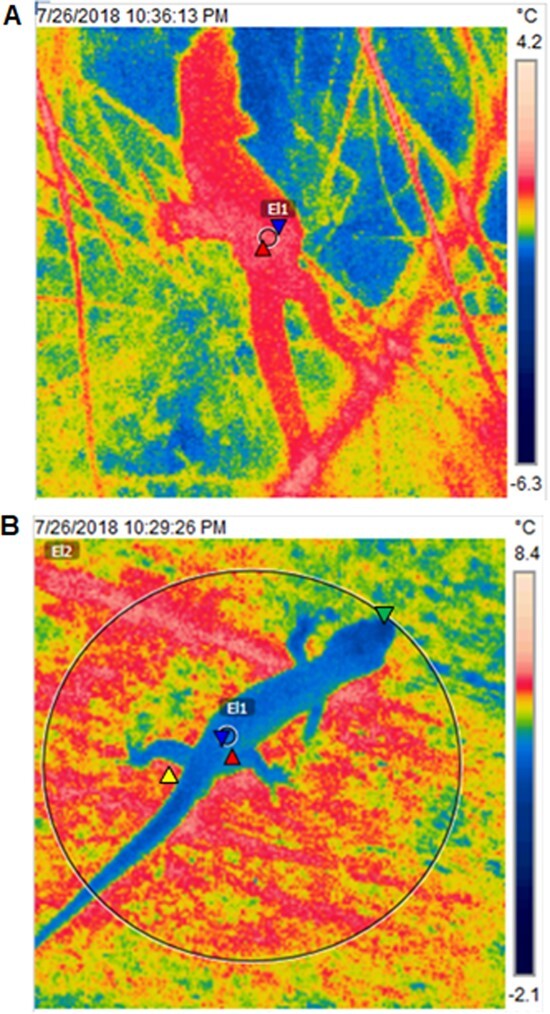
Thermograms of *Woodworthia* ‘Otago/Southland’ geckos measured during nighttime field *T_b_* surveys at Macraes, Otago, New Zealand. (A) The lowest field *T_b_* observed (EI1: mean 1.4°C for a nonpregnant female gecko found on vegetation). (B) A male gecko captured at the base of a rock tor that had a residual surface temperature (EI2: 6.3°C; yellow triangle by the tail of the gecko denotes the warmest spot on the rock tor) higher than the gecko’s body temperature (EI1: mean 3.8°C). The circles (big, EI2 and small, EI1) represent the ROIs, the red and yellow triangles are the hottest, while blue and green triangles are the coldest spots within the ROI, respectively. The small circular ROI on the geckos’dorsum corresponded to the circumference of the sensor of a mini-infrared thermometer (Chukwuka *et al.,* 2019).

**Figure 5:**
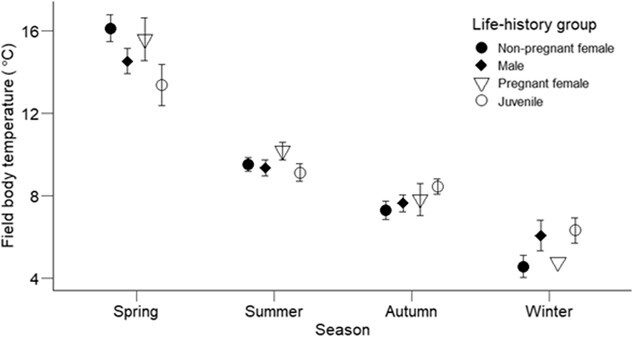
Mean ± SE nighttime field body temperature of emerged geckos (*Woodworthia* ‘Otago/Southland’) measured within five hours after dusk at Macraes, Otago, New Zealand. The skin surface temperature of the geckos varied with the season (*P* = 0.0001) but did not differ significantly among life history groups (*P* = 0.12). The higher field body temperatures in spring compared to summer are explained by warmer air temperature on the sampling nights. Sample sizes for male, non-pregnant female, pregnant female and juveniles were as follows: Spring: 35, 15, 14 and 12, respectively; Summer: 37, 26, 21 and 27, respectively; Autumn: 23, 21, 6 and 21, respectively; and Winter: 12, 12, 1 and 14, respectively.

The highest nighttime field *T_b_* when emerged was recorded in spring for a pregnant female (22.6°C) and the lowest in winter for a non-pregnant female (1.4°C; Figure 4A). Most of the captured geckos were found motionless either on vegetation or a rock tor. Juveniles were sometimes present at high numbers (up to 16 juveniles) within the shelter of a golden spaniard plant (a type of spear grass, *Aciphylla aurea*). Mean field *T_b_* differed significantly across the seasons (χ^2^ = 446.20, *df* = 3, *P* < 0.001), being lower in summer compared to spring, and with time after dusk (χ^2^ = 5.76, *df* = 1, *P* = 0.01; Figure 5). The unexpectedly higher values in spring than summer are explained by warmer air temperature on the sampling nights. However, the effect of life history group on mean field *T_b_* was not statistically significant (χ^2^ = 5.84, *df* = 3, *P* = 0.12), and nor was there an interaction between life history group and season (χ^2^ = 14.20, *df* = 9, *P* = 0.11).

**Figure 6:**
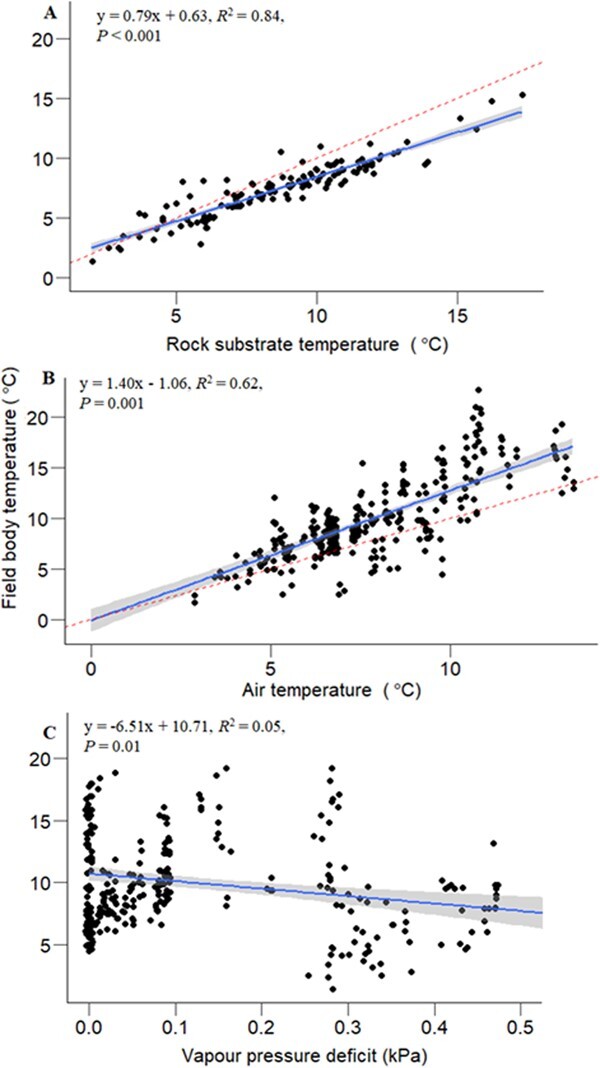
Relationships between nighttime active field body temperature (*T_b_*) of the gecko *Woodworthia* ‘Otago/Southland’ and environmental variables at Macraes, Otago, New Zealand. (A) Relationships with the rock substrate temperature on rock tors where the geckos were captured at night. Rock substrate temperature was measured with a thermal infrared camera. Substrate temperature was statistically warmer than the gecko body temperatures (*P* < 0.001). (B) Field *T_b_* increased with increasing air temperature and was statistically warmer than the air temperature. (C) Field *T_b_* decreased as air become drier (increased VPD). The air temperature was measured concurrently with the field *T_b_*, with a Themocron iButton hung at 1 m above the ground. VPD was calculated from the air temperature above, and relative humidity data measured every hour at a nearby weather station at Macraes corresponding to the time when the field sampling was done (obtained from Fire and Emergency Services New Zealand). The grey-shaded area is the 95% confidence interval of the regression line. The broken red lines represent the isothermal line.

Nighttime field *T_b_* (mean: 7.41 ± 0.10°C) was significantly lower than rock surface temperature measured from the same spot where geckos were captured at night (mean: 8.58 ± 0.02°C; χ^2^ = 637.45, *df* = 1, *P* < 0.001; Figure 4B), with a strong positive relationship (*R^2^* = 0.84, *P* < 0.001; Figure 6A). In addition, there was a positive effect of air temperature (mean air temperature: 6.56 ± 0.001°C) and VPD (mean VPD: 0.13 ± 0.84 kPa) on the geckos’ nighttime field *T_b_* (air temperature: *F_1,286_* = 613.84, *P* < 0.001; VPD: *F_1,286_* = 61.27, *P* < 0.001; Figure 6B,C), but no effect of wind speed (mean: 3.8 ± 0.08 m/s; *F_1,284_* = 0.12, *P* = 0.72). The overall effect of thermal variables on field *T*_b_ is that the mean nighttime field *T*_b_ is intermediate between the (warmer) rock surface and the (cooler) night air temperature. The effect of air temperature came close to being influenced by VPD (air temperature: VPD: *F_1,284_* = 3.35, *P* = 0.06; but not wind speed (air temperature: wind speed: *F_1,284_* = 0.30, *P* = 0.58). The effect of wind speed on nighttime field body temperature was influenced by the drying power of the air (wind speed: VPD, *F_1,284_* = 7.28, *P* = 0.007).

## Discussion

Weather conditions at the time of sampling influenced the nighttime emergence of Otago/Southland geckos, with high emergence activity on warm nights. Operative environmental temperature (*T_e_*) and emergence activity were high when air temperature was high and wind speed low. The number of emerged geckos sighted at night and the duration of emergence were highest in spring and summer (with *T_b_* up to 22.63°C in spring) and continued (at low levels) during autumn and winter despite the cold climate. We also found that Otago/Southland geckos are active at night with field body temperature (*T_b_*) as low as 1.4°C when the air temperature was less than 1°C in winter. At night, the maximum *T_e_* was below the gecko’s preferred body temperature in all seasons (Rock *et al.,* 2000).

Nocturnal activity in our study species is interpreted as a function of temperature, and to an extent, time after dusk. Although activity of lizards, in general, is associated with temperature (Huey, 1982), activity at such a low air temperature (<2°C) is peculiar for mid-alpine Otago/Southland geckos (herein) and probably also for another New Zealand species, the orange-spotted gecko (*Mokopirirakau* ‘Roys Peak’), which was found active in the alpine zone, with lizard model temperatures averaging as low as −0.8°C (Bertoia *et al.,* 2021). In an Australian nocturnal gecko, *Gehyra variegata*, activity was high on warm nights (air temperature above 20°C) and dropped sharply on cold nights with air temperature below 18°C (Bustard, 1967). Activity at low nighttime temperatures may be costly for the lizards because low locomotory speed at a lower temperature would increase the risk of predation by endotherms (Cooper, 2000) and reduce the likelihood of prey capture (Hare *et al.,* 2007; Gaby *et al.,* 2011). To counter locomotory performance at low temperatures, nocturnal lizards may also have evolved a low minimal cost of locomotion by reducing muscle force and mechanical work (Farley and Emshwiller, 1996; Hare *et al.,* 2007), and enhanced mitochondrial functions (Autumn *et al.,* 1999). However, whether increased nighttime activity on warm nights in this study poses a predation risk to this species and other nocturnal lizards at high latitudes remains unclear. At high latitudes, it is likely that lizards are at less risk of predation by introduced mammalian predators (currently their main predators) at warmer night-time temperatures than at cooler temperatures when locomotion may be impaired. So, the consequences of increased nocturnal activity under climate change will probably depend both on how climate change affects mammal populations (e.g., allowing some species to become residents, and others to be in greater abundance, at higher elevations; O'Donnell *et al.,* 2017) and on lizard biology (e.g., greater nighttime emergence (this study) and potentially also increased reproduction; Cree and Hare, 2016a).

High wind speeds reduced the nighttime emergence of Otago/Southland geckos; most sightings of emerged geckos occurred at low wind speeds. In studies on other lizards, wind facilitated the cooling of field *T*_b_ through convection and may lead to a drop in body temperature when out of the retreat (Maia-Carneiro *et al.,* 2012; Gontijo *et al.,* 2018). In contrast, activity at high wind speed has been inferred for the diurnal lizard, *Anolis lemurinus* (Logan *et al.,* 2015) but wind significantly reduced thermoregulatory set-point temperatures of a New Zealand diurnal skink in a controlled experiment (Virens and Cree, 2022). A previous report on the activity of *Woodworthia maculata* (then described as *Hoplodactylus maculatus*) on Takapourewa/Stephens Island in New Zealand observed relatively low activity on nights with high wind speed (Walls, 1983). Due to moderate elevation (~600 m) where our study population thrives, gale-force winds can interrupt lizard activity at night even if other weather factors such as temperature are favourable. As a consequence of wind, more body water may be lost due to evaporation from the integument (Waldschmidt and Porter, 1987), and communication between individuals will be impaired (Peters *et al.,* 2007). High windspeed counters the thermoregulatory processes, decreases thermal accuracy and increases desiccation rates (Ortega *et al.,* 2017).

Otago/Southland geckos were found active over a broad range of temperatures with lowest *T_b_* of 1.4°C, which to our knowledge is the lowest field *T_b_* ever measured for an active lizard in New Zealand (Hare and Cree, 2016) or elsewhere in the world (Meiri *et al.,* 2013). These geckos were either on vegetation or motionless on rock tors with rock surface temperature that was typically higher than the air temperature at low nighttime air temperatures. In our study, field *T_b_* is in general intermediate between *T*_a_ and rock temperature, hinting that the geckos may have the opportunity to thermoregulate to a small extent (Nordberg and Schwarzkopf, 2019), by where they position themselves at night (because the tors store heat and there is thermal heterogeneity among the available microhabitats). The low night field *T_b_* in this study indicated that Otago/Southland geckos are capable of being active at an air temperature of less than 2°C, well below their physiological optimum (Gaby *et al.,* 2011). Most of the geckos captured at night were sluggish and were unable to move rapidly even after taking the body temperature measurement, suggesting that Otago/Southland geckos are ‘sit-and-wait’ ambush feeders, passively waiting for prey and capturing them without chase or pursuit (Spencer and Grimmond, 1994) as a result of low locomotory performance at low ambient temperature (Hare *et al.,* 2007). In winter, a season when these geckos had been thought to be inactive/torpid (Hare and Cree, 2016), field sampling on 12 rock tors (nighttime air temperature: ~4°C) yielded up to 26 adult geckos active, and more than 18 juveniles were found on a golden spaniard (*A. aurea*). These juveniles may have been deriving a thermal benefit from the shelter provided by the speargrass, as well as foraging while avoiding predation.

Activity at such a low *T_b_* in the field was unexpected, although laboratory studies show that Otago/Southland geckos are capable of righting themselves at least as low as a *T_b_* of 0.8°C when the air temperature was −0.5°C (Besson and Cree, 2010). They have also been seen active at a room temperature of 5°C in the laboratory (Vermunt *et al.,* 2014). Activity at low nighttime body temperature in our field study may have been enhanced by high daytime temperature prior to the sampled nights (Walls, 1983), but clearly, the geckos are capable of functioning at low air and body temperatures. At low *T_b_*, movement and escape from predators are impaired (Bennett, 1980) due to reduced sprint speed (Gaby *et al.,* 2011). Geckos may have emerged from deep crevices with more stable temperature conditions (Chukwuka, 2020), or they can tolerate low body temperatures (Bertoia *et al.,* 2021) with a low cost of locomotion (Hare *et al.,* 2007) and high aerobic capacity (Hare *et al.,* 2010), though probably not allowing their body water to freeze (Claussen *et al.,* 1990; Costanzo *et al.,* 1995). The survival mechanisms at low ambient temperature, including whether Otago/Southland geckos are freeze-tolerant, remain unknown. We suggest further research to understand the physiological mechanisms at low temperatures in *Woodworthia* geckos and other New Zealand lizards living in similar cold microhabitats.

High-latitude lizards live in a heterogeneous thermal environment in which microclimate temperatures are frequently lower than the voluntary thermal maximum (Chukwuka *et al.,* 2021). Thus, cool-climate lizards may be at less immediate risk than their tropical counterparts from overheating due to global climate change (Deutsch *et al.,* 2008; Clarke and Zani, 2012; Huey *et al.,* 2012), although some microhabitats even within retreats have the potential to exceed VT_max_ on hot summer days (Chukwuka *et al.,* 2020). Warmer conditions will enhance activity for high-latitude lizards when *T_e_* frequently remains below the optimum temperature (*T_o_*), while the animal frequently explores the heterogenous microhabitat (Huey *et al.,* 2012). In terms of activity, high-latitude lizards may initially benefit from future climate heating until field-active *T_b_* exceeds the optimum temperature (Huey *et al.,* 2012). Currently, high-latitude lizards possess higher thermal safety margins, with lower active body temperature, critical thermal minimum and selected temperatures, and broader optimal temperature ranges (Besson and Cree, 2010; Gaby *et al.,* 2011; Hare and Cree, 2016) compared to lower-latitude species (Huey, 1982; Clusella-Trullas *et al.,* 2011; Sunday *et al.,* 2014). Thus, mean activity levels for cool-temperate reptiles and amphibians will initially increase as climates warm (Buckley *et al.,* 2012; Huang *et al.,* 2013; Gade *et al.,* 2020).

In New Zealand, future climate projections under representative concentration pathway 4.5 (a relatively optimistic scenario for greenhouse gas emissions) suggest that the region within which our Macraes field site falls will experience warmer temperatures with more extremely hot days (days with air temperature > 30°C) in 2040 and 2090, reduced rainfall in summer and increased rainfall in winter and spring, decreased wind conditions by an average of −2.5% in 2040 and −1.5% in 2090 and a reduced number of frosty days (Macara *et al.,* 2019). Winter temperatures are predicted to warm more rapidly than summer temperatures in the same year (Macara *et al.,* 2019). Warmer night temperature, together with seasonal changes in rainfall pattern and reduced wind speed (with reduced cooling of the rock substrate), have the potential to increase the nocturnal activity of Otago/Southland geckos. The increase in nighttime air temperature is predicted to persist over the winter season resulting in a probable increase in mid-winter nocturnal activity (herein), allowing geckos to forage and find mates more effectively. Warmer temperatures may also present growth and reproductive benefits (Kubisch *et al.,* 2012; Cree and Hare, 2016a). However, the long-term consequences of increased climate heating (including the potential for harmful extremes of temperature) on activity timing, increase in winter rainfall and other important aspects of natural history including maternal gestation length and offspring phenotypic traits, remain unclear. Recent finding suggested that lizards at higher elevation will experience greater impact of climate change due to decrease in habitat availability especially for our study species (Jarvie *et al.,* 2022); but consequences of increased warming may not be enormous when microclimate properties and lizards’ biology are considered (Chukwuka *et al.,* 2021). In addition, the potential for increased vulnerability to predation while emerging (Wilson and Cooke, 2004; Sperry *et al.,* 2010), including from introduced mammals, which themselves will be impacted by climate change, will be important to address.

## Funding

The study was supported by PhD grant (2016) from Department of Zoology, University of Otago, and Miss E. L. Hellaby Indigenous Grasslands Research Trust research grant (2017) to Christian Chukwuka.

## Data Availability Statement

The data used in this article is available on request to the corresponding author.

## Supplementary Material

Web_Material_coac082

## References

[ref1] Adolph SC , Porter WP (1993) Temperature, activity, and lizard life histories. Am Nat 142: 273–295. 10.1086/285538. 19425979

[ref2] Angilletta, M. J. (2009) Thermal Adaptation: A Theoretical and Empirical Synthesis. Oxford University Press, USA10.1093/acprof:oso/9780198570875.001.1.

[ref3] Anholt BR , Werner E, Skelly DK (2000) Effect of food and predators on the activity of four larval ranid frogs. Ecology 81: 3509–3521. 10.1890/0012-9658(2000)081[3509:EOFAPO]2.0.CO;2.

[ref4] Autumn K , Farley CT, Emshwiller M, Full RJ (1997) Low cost of locomotion in the banded gecko: a test of the nocturnality hypothesis. Physiol Zool 70: 660–669. 10.1086/515880. 9361140

[ref5] Autumn K , Jindrich D, DeNardo D, Mueller R (1999) Locomotor performance at low temperature and the evolution of nocturnality in geckos. Evolution 53: 580–599. 10.1111/j.1558-5646.1999.tb03793.x. 28565430

[ref6] Autumn K , Weinstein RB, Full RJ (1994) Low cost of locomotion increases performance at low temperature in a nocturnal lizard. Physiol Zool 67: 238–262. 10.1086/physzool.67.1.30163845.

[ref7] Bakken GS (1992) Measurement and application of operative and standard operative temperatures in ecology. Am Zool 32: 194–216. 10.1093/icb/32.2.194.

[ref8] Bennett AF (1980) The thermal dependence of lizard behaviour. Anim Behav 28: 752–762. 10.1016/S0003-3472(80)80135-7.

[ref9] Bertoia A , Monks J, Knox C, Cree A (2021) A nocturnally foraging gecko of the high-latitude alpine zone: extreme tolerance of cold nights, with cryptic basking by day. J Therm Biol 99: 102957. 10.1016/j.jtherbio.2021.102957. 34420613

[ref10] Besson AA , Cree A (2010) A cold-adapted reptile becomes a more effective thermoregulator in a thermally challenging environment. Oecologia 163: 571–581. 10.1007/s00442-010-1571-y. 20140685

[ref11] Booth JG , Casella G, Friedl H, Hobert JP (2003) Negative binomial loglinear mixed models. *Stat Modelling* 3: 179–191. 10.1191/1471082X03st058oa.

[ref12] Brooks ME , Kristensen K, Darrigo MR, Rubim P, Uriarte M, Bruna E, Bolker BM (2019) Statistical modeling of patterns in annual reproductive rates. Ecology 100: e02706–e02707. 10.1002/ecy.2706. 30916779

[ref13] Brooks ME , Kristensen K, van Benthem KJ, Magnusson A, Berg CW, Nielsen A, Skaug HJ, Mächler M, Bolker BM (2017) glmmTMB balances speed and flexibility among packages for zero-inflated generalized linear mixed modeling. The R Journal 9: 378–400. 10.32614/RJ-2017-066.

[ref14] Brown GP , Shine R (2002) Influence of weather conditions on activity of tropical snakes. Austral Ecol 27: 596–605. 10.1046/j.1442-9993.2002.01218.x.

[ref15] Buckley LB , Hurlbert AH, Jetz W (2012) Broad-scale ecological implications of ectothermy and endothermy in changing environments. Glob Ecol Biogeogr 21: 873–885. 10.1111/j.1466-8238.2011.00737.x.

[ref16] Bustard HR (1967) Activity cycle and thermoregulation in the Australian gecko *Gehyra variegata*. Copeia 1967: 753–758. 10.2307/1441885.

[ref17] Chukwuka CO (2020) Microhabitat use by the nocturnal, cool-climate gecko Woodworthia “Otago/Southland” in the context of global climate change. PhD thesis,. University of Otago, Dunedin, New Zealand

[ref18] Chukwuka CO , Mello RSR, Cree A, Monks JM (2021) Thermal heterogeneity of selected retreats in cool-temperate viviparous lizards suggests a potential benefit of future climate warming. J Therm Biol: 97: 102869. 10.1016/j.jtherbio.2021.102869. 33863433

[ref19] Chukwuka CO , Monks JM, Cree A (2020) Heat and water loss versus shelter: a dilemma in thermoregulatory decision making for a retreat-dwelling nocturnal gecko. J Exp Biol 223: jeb231241. 10.1242/jeb.231241. 32778565

[ref20] Chukwuka CO , Virens J, Cree A (2019) Accuracy of an inexpensive, compact infrared thermometer for measuring skin surface temperature of small lizards. J Therm Biol 84: 285–291. 10.1016/j.jtherbio.2019.07.016. 31466766

[ref21] Clarke DN , Zani PA (2012) Effects of night-time warming on temperate ectotherm reproduction: potential fitness benefits of climate change for side-blotched lizards. J Exp Biol 215: 1117–1127. 10.1242/jeb065359. 22399656

[ref22] Claussen DL , Townsley MD, Bausch RG (1990) Supercooling and freeze-tolerance in the European wall lizard, *Podarcis muralis,* with a revisional history of the discovery of freeze-tolerance in vertebrates. J Comp Physiol B 160: 137–143. 10.1007/BF00300945.

[ref23] Clusella-Trullas S , Blackburn TM, Chown SL (2011) Climatic predictors of temperature performance curve parameters in ectotherms imply complex responses to climate change. *Am Nat* 177: 738–751. 10.1086/660021. 21597251

[ref24] Cooper WE (2000) Effect of temperature on escape behaviour by an ectothermic vertebrate, the keeled earless lizard *(Holbrookia propinqua*). Behaviour 137: 1299–1315. 10.1163/156853900501935.

[ref25] Costanzo JP , Grenot C, Lee RE (1995) Supercooling, ice inoculation and freeze tolerance in the European common lizard, *Lacerta vivipara*. J Comp Physiol B 165: 238–244. 10.1007/BF00260815. 7665737

[ref26] Cox DTC , Maclean IMD, Gardner AS, Gaston KJ (2020) Global variation in diurnal asymmetry in temperature, cloud cover, specific humidity and precipitation and its association with leaf area index. Glob Chang Biol 26: 7099–7111. 10.1111/gcb.15336. 32998181

[ref27] Cree A , Hare KM (2016a) Maternal basking regime has complex implications for birthdate and offspring phenotype in a nocturnally foraging, viviparous gecko. J Exp Biol 219: 2934–2943. 10.1242/jeb.140020. 27436138

[ref28] Cree, A., and K. M. Hare. (2016b) Reproduction and life history of New Zealand lizards. In D. G. Chapple, eds, New Zealand lizards, pp. 169–206. Springer, Switzerland, 10.1007/978-3-319-41674-8_7.

[ref29] Crowley SR (1987) The effect of desiccation upon the preferred body temperature and activity level of the lizard *Sceloporus undulatus*. Copeia 1987: 25–32. 10.2307/1446033.

[ref30] Deutsch CA , Tewksbury JJ, Huey RB, Sheldon KS, Ghalambor CK, Haak DC, Martin PR (2008) Impacts of climate warming on terrestrial ectotherms across latitude. Proc Natl Acad Sci U S A 105: 6668–6672. 10.1073/pnas.0709472105. 18458348 PMC2373333

[ref31] Dzialowski EM (2005) Use of operative temperature and standard operative temperature models in thermal biology. J Therm Biol 30: 317–334. https://doi.org/10.1016/j.jtherbio.2005.01.005.

[ref32] Farley C , Emshwiller M (1996) Efficiency of uphill locomotion in nocturnal and diurnal lizards. J Exp Biol 199: 587–592. https://doi.org/10.1242/jeb.199.3.587. 9318297 10.1242/jeb.199.3.587

[ref33] Gaby MJ , Besson AA, Bezzina CN, Caldwell AJ, Cosgrove S, Cree A, Haresnape S, Hare KM (2011) Thermal dependence of locomotor performance in two cool-temperate lizards. J Comp Physiol A 197: 869–875. 10.1007/s00359-011-0648-3. 21547573

[ref34] Gade MR , Connette GM, Crawford JA, Hocking DJ, Maerz JC, Milanovich JR, Peterman WE (2020) Predicted alteration of surface activity as a consequence of climate change. Ecology 101: e03154. 10.1002/ecy.3154. 32740923

[ref35] Gaston KJ (2019) Nighttime ecology: the “nocturnal problem” revisited. Am Nat 193: 481–502. 10.1086/702250. 30912975

[ref36] Gibson S , Penniket S, Cree A (2015) Are viviparous lizards from cool climates ever exclusively nocturnal? Evidence for extensive basking in a New Zealand gecko. Biol J Linn Soc 115: 882–895. 10.1111/bij.12533.

[ref37] Gontijo ASB , Garcia CS, Righi AF, Galdino CAB (2018) To warm on the rocks, to cool in the wind: thermal relations of a small-sized lizard from a mountain environment. J Therm Biol 76: 52–57. 10.1016/j.jtherbio.2018.07.003. 30143297

[ref38] Gordon CE , Dickman CR, Thompson MB (2010) What factors allow opportunistic nocturnal activity in a primarily diurnal desert lizard (*Ctenotus pantherinus*)? Comp Biochem Physiol A Mol Integr Physiol 156: 255–261. 10.1016/j.cbpa.2010.02.007. 20170741

[ref39] Greenberg S (2017) Timelapse2: An image analyser for camera traps. Available at http://saul.cpsc.ucalgary.ca/timelapse/pmwiki.php?n=Main.HomePage.

[ref40] Gunderson AR , Leal M (2016) A conceptual framework for understanding thermal constraints on ectotherm activity with implications for predicting responses to global change. Ecol Lett 19: 111–120. 10.1111/ele.12552. 26647860

[ref41] Hare, K. M., D. G. Chapple, D. R. Towns, and D. van Winkel. 2016. The ecology of New Zealand’s lizards. In D. G. Chapple, ed, New Zealand Lizards, pp. 239–267. Springer, Switzerland, 10.1007/978-3-319-41674-8_9.

[ref42] Hare, K. M., and A. Cree. 2016. Thermal and metabolic physiology of New Zealand lizards. In D. G. Chapple, ed, New Zealand Lizards, pp. 239–267. Springer, Switzerland, 10.1007/978-3-319-41674-8_9.

[ref43] Hare KM , Miller JH, Clark AG, Daugherty CH (2005) Total lactate dehydrogenase activity of tail muscle is not cold-adapted in nocturnal lizards from cool-temperate habitats. Comp Biochem Physiol Part B Biochem Mol Biol 142: 438–444. 10.1016/j.cbpb.2005.09.003. 16242367

[ref44] Hare KM , Pledger S, Thompson MB, Miller JH, Daugherty CH (2007) Low cost of locomotion in lizards that are active at low temperatures. Physiol Biochem Zool 80: 46–58. 10.1086/509237. 17160879

[ref45] Hare KM , Pledger S, Thompson MB, Miller JH, Daugherty CH (2010) Nocturnal lizards from a cool-temperate environment have high metabolic rates at low temperatures. J Comp Physiol B 180: 1173–1181. 10.1007/s00360-010-0489-3. 20559839

[ref46] Hitchmough RA , Barr B, Knox C, Lettink M, Monks JM, Patterson GB, Reardon JT, van Winkel D, Rolfe J, Michel P (2021) Conservation Status of New Zealand Reptiles. Wellington, Department of Conservation, p. 27

[ref47] Hobbs MT , Brehme CS (2017) An improved camera trap for amphibians, reptiles, small mammals, and large invertebrates. PLoS One 12: e0185026. 10.1371/journal.pone.0185026. 28981533 PMC5628828

[ref48] Huang S-P , Chiou C-R, Lin T-E, Tu M-C, Lin C-C, Porter WP (2013) Future advantages in energetics, activity time, and habitats predicted in a high-altitude pit viper with climate warming. *Funct Ecol* 27: 446–458. 10.1111/1365-2435.12040.

[ref49] Huey RB (1982) Temperature, physiology, and the ecology of reptiles. In C Gans, FH Pough, eds, Biology of the Reptilia. Academic Press, New York, New York, pp. 25–91

[ref50] Huey RB , Kearney MR, Krockenberger A, Holtum JA, Jess M, Williams SE (2012) Predicting organismal vulnerability to climate warming: roles of behaviour, physiology and adaptation. Phil Trans R Soc B 367: 1665–1679. 10.1098/rstb.2012.0005. 22566674 PMC3350654

[ref51] Huey RB , Niewiarowski PH, Kaufmann J, Herron JC (1989) Thermal biology of nocturnal ectotherms: is sprint performance of geckos maximal at low body temperatures? Physiol Zool 62: 488–504. 10.1086/physzool.62.2.30156181.

[ref52] Huey RB , Tewksbury JJ (2009) Can behavior douse the fire of climate warming? Proc Natl Acad Sci 106: 3647–3648. 10.1073/pnas.0900934106. 19276126 PMC2656133

[ref53] IPCC (2021) In V Masson-Delmotte, P Zhai, A Pirani, SL Connors, C Péan, S Berger, N Caud, Y Chen, L Goldfarb, MI Gomis et al., eds, Climate Change 2021: The Physical Science Basis. Contribution of Working Group I to the Sixth Assessment Report of the Intergovernmental Panel on Climate Change. Cambridge University Press, Cambridge, United Kingdom and New York, NY, USA.

[ref54] James RS (2013) A review of the thermal sensitivity of the mechanics of vertebrate skeletal muscle. J Comp Physiol B 183: 723–733. 10.1007/s00360-013-0748-1. 23483325

[ref55] Jarvie S , Ingram T, Chapple DG, Hitchmough RA, Nielsen SV, Monks JM (2022) Variable vulnerability to climate change in New Zealand lizards. J Biogeogr 49: 431–442. 10.1111/jbi.14314.

[ref56] Johansson F , Orizaola G, Nilsson-Örtman V (2020) Temperate insects with narrow seasonal activity periods can be as vulnerable to climate change as tropical insect species. Sci Rep 10: 8822. 10.1038/s41598-020-65608-7. 32483233 PMC7264184

[ref57] Jucker T , Hardwick SR, Both S, Elias DMO, Ewers RM, Milodowski DT, Swinfield T, Coomes DA (2018) Canopy structure and topography jointly constrain the microclimate of human-modified tropical landscapes. Glob Chang Biol 24: 5243–5258. 10.1111/gcb.14415. 30246358

[ref58] Kearney MR , Munns SL, Moore D, Malishev M, Bull CM (2018) Field tests of a general ectotherm niche model show how water can limit lizard activity and distribution. *Ecol Monogr* 88: 672–693. 10.1002/ecm.1326.

[ref59] Kearney MR , Porter W (2009) Mechanistic niche modelling: combining physiological and spatial data to predict species’ ranges. Ecol Lett 12: 334–350. 10.1111/j.1461-0248.2008.01277.x. 19292794

[ref60] Kubisch E , Piantoni C, Williams J, Scolaro A, Navas CA, Ibargüengoytía NR (2012) Do higher temperatures increase growth in the nocturnal gecko *Homonota darwini* (Gekkota: Phyllodactylidae)? A skeletochronological assessment analyzed at temporal and geographic scales. J Herpetol 46: 587–595. 10.1670/10-277.

[ref61] Lettink M , Monks JM (2016) Survey and monitoring methods for New Zealand lizards. *J R Soc N Z* 46: 16–28. 10.1080/03036758.2015.1108343.

[ref62] Logan ML , Fernandez SG, Calsbeek R (2015) Abiotic constraints on the activity of tropical lizards. *Funct* *Ecol* 29: 694–700. 10.1111/1365-2435.12379.

[ref63] López-Alcaide S , González-Salazar C, Macip-Ríos R, Martínez-Meyer E (2017) Using microhabitat thermal heterogeneity to avoid lethal overheating: an empirical approximation in reproductive oviparous and viviparous lizards. *Rev Mex Biodivers* 88: 683–690. 10.1016/j.rmb.2017.07.005.

[ref64] Lorenzon P , Clobert J, Oppliger A, John-Alder H (1999) Effect of water constraint on growth rate, activity and body temperature of yearling common lizard (*Lacerta vivipara*). Oecologia 118: 423–430. 10.1007/s004420050744. 28307409

[ref65] Macara G , Woolley J-M, Zammit C, Pearce P, Stuart S, Wadhwa S, Sood A, Collins D (2019) In N. I. o. W. A. R. , ed, Climate Change Projections for the Otago Region, Ltd., NIWA, Wellington, pp. 21–55

[ref66] Maia-Carneiro T , Dorigo TA, Rocha CFD (2012) Influences of seasonality, thermal environment and wind intensity on the thermal ecology of Brazilian sand lizards in a restinga remnant. S Am J Herpetol 7: 241–251. 10.2994/057.007.0306.

[ref67] Martin TG , Wintle BA, Rhodes JR, Kuhnert PM, Field SA, Low-Choy SJ, Tyre AJ, Possingham HP (2005) Zero tolerance ecology: improving ecological inference by modelling the source of zero observations. Ecol Lett 8: 1235–1246. 10.1111/j.1461-0248.2005.00826.x. 21352447

[ref68] Meiri S , Bauer AM, Chirio L, Colli GR, Das I, Doan TM, Feldman A, Herrera F-C, Novosolov M, Pafilis P et al. (2013) Are lizards feeling the heat? A tale of ecology and evolution under two temperatures. Glob Ecol Biogeogr 22: 834–845. 10.1111/geb.12053.

[ref69] Nielsen SV , Bauer AM, Jackman TR, Hitchmough RA, Daugherty CH (2011) New Zealand geckos (Diplodactylidae): cryptic diversity in a post-Gondwanan lineage with trans-Tasman affinities. Mol Phylogenet Evol 59: 1–22. 10.1016/j.ympev.2010.12.007. 21184833

[ref70] Nordberg EJ , Schwarzkopf L (2019) Heat seekers: a tropical nocturnal lizard uses behavioral thermoregulation to exploit rare microclimates at night. J Therm Biol 82: 107–114. 10.1016/j.jtherbio.2019.03.018. 31128638

[ref71] O'Donnell CFJ , Weston KA, Monks JM (2017) Impacts of introduced mammalian predators on New Zealand’s alpine fauna. N Z J Ecol 41: 1–22. 10.20417/nzjecol.41.18.

[ref72] Ortega Z , Mencía A, Pérez-Mellado V (2017) Wind constraints on the thermoregulation of high mountain lizards. Int J Biometeorol 61: 565–573. 10.1007/s00484-016-1233-9. 27528186

[ref73] Paladino FV (1985) Temperature effects on locomotion and activity bioenergetics of amphibians, reptiles, and birds. Am Zool 25: 965–972. 10.1093/icb/25.4.965.

[ref74] Penniket S , Cree A (2015) Adherence to Bergmann's rule by lizards may depend on thermoregulatory mode: support from a nocturnal gecko. Oecologia 178: 427–440. 10.1007/s00442-015-3239-0. 25663371

[ref75] Peters RA , Hemmi JM, Zeil J (2007) Signaling against the wind: modifying motion-signal structure in response to increased noise. Curr Biol 17: 1231–1234. 10.1016/j.cub.2007.06.035. 17614279

[ref76] R Core Team (2008) R: A Language and Environment for Statistical Computing. R Foundation for Statistical Computing, Vienna, Austria

[ref77] Rock J , Andrews RM, Cree A (2000) Effects of reproductive condition, season, and site on selected temperatures of a viviparous gecko. Physiol Biochem Zool 73: 344–355. 10.1086/316741. 10893174

[ref78] Rock J , Cree A, Andrews RM (2002) The effect of reproductive condition on thermoregulation in a viviparous gecko from a cool climate. J Therm Biol 27: 17–27. 10.1016/S0306-4565(01)00011-0.

[ref79] Sawilowsky SS (2009) New effect size rules of thumb. J Mod Appl Stat Methods 8: 597–599. 10.22237/jmasm/1257035100.

[ref80] Shine R , Kearney M (2001) Field studies of reptile thermoregulation: how well do physical models predict operative temperatures? Funct Ecol 15: 282–288. 10.1046/j.1365-2435.2001.00510.x.

[ref81] Sinervo B , Mendez-de-la-Cruz F, Miles DB, Heulin B, Bastiaans E, Villagran-Santa Cruz M, Lara-Resendiz R, Martinez-Mendez N, Calderon-Espinosa ML, Meza-Lazaro RN et al. (2010) Erosion of lizard diversity by climate change and altered thermal niches. Science 328: 894–899. 10.1126/science.1184695. 20466932

[ref82] Sound P , Veith M (2000) Weather effects on intrahabitat movements of the western green lizard, *Lacerta bilineata* (Daudin, 1802), at its northern distribution range border: a radio-tracking study. Can J Zool 78: 1831–1839. 10.1139/z00-103.

[ref83] Spencer NJ , Grimmond NM (1994) Influence of elevation on the thermoregulation of two sympatric lizards. N Z J Zool 21: 379–385. 10.1080/03014223.1994.9518007.

[ref84] Sperry JH , Blouin-Demers G, Carfagno GLF, Weatherhead PJ (2010) Latitudinal variation in seasonal activity and mortality in ratsnakes (*Elaphe obsoleta*). Ecology 91: 1860–1866. 10.1890/09-1154.1. 20583726

[ref85] Sperry JH , Ward MP, Weatherhead PJ (2013) Effects of temperature, moon phase, and prey on nocturnal activity in ratsnakes: an automated telemetry study. J Herpetol 47: 105–111. 10.1670/11-325.

[ref86] Storey KB (2006) Reptile freeze tolerance: metabolism and gene expression. Cryobiology 52: 1–16. 10.1016/j.cryobiol.2005.09.005. 16321368

[ref87] Stroud JT , Mothes CC, Beckles W, Heathcote RJP, Donihue CM, Losos JB (2020) An extreme cold event leads to community-wide convergence in lower temperature tolerance in a lizard community. Biol Lett 16: 20200625. 10.1098/rsbl.2020.0625. 33081602 PMC7655475

[ref88] Sunday JM , Bates AE, Kearney MR, Colwell RK, Dulvy NK, Longino JT, Huey RB (2014) Thermal-safety margins and the necessity of thermoregulatory behavior across latitude and elevation. Proc Natl Acad Sci 111: 5610–5615. 10.1073/pnas.1316145111. 24616528 PMC3992687

[ref89] Tewksbury JJ , Huey RB, Deutsch CA (2008) Putting the heat on tropical animals. Science 320: 1296–1297. 10.1126/science.1159328. 18535231

[ref90] Vermunt A , Hare KM, Besson AA (2014) Unusual change in activity pattern at cool temperature in a reptile (*Sphenodon punctatus*). J Therm Biol 42: 40–45. 10.1016/j.jtherbio.2014.02.021. 24802147

[ref91] Virens E , Cree A (2022) Wind of change: a diurnal skink thermoregulates between cooler set-points and for an increased amount of time in the presence of wind. J Exp Biol 225: jeb.244038. 10.1242/jeb.244038. PMC9001919 35179605

[ref92] Vitt LJ , Pianka ER, Cooper WE Jr, Schwenk K (2003) History and the global ecology of squamate reptiles. Am Nat 162: 44–60. 10.1086/375172. 12856236

[ref93] Waldschmidt SR , Porter WP (1987) A model and experimental test of the effect of body temperature and wind speed on ocular water loss in the lizard, *Uta stansburiana*. Physiol Zool 60: 678–686. 10.1086/physzool.60.6.30159982.

[ref94] Walls GY (1983) Activity of the tuatara and its relationships to weather conditions on Stephens Island, Cook Strait, with observations on geckos and invertebrates. N Z J Zool 10: 309–317. 10.1080/03014223.1983.10423920.

[ref95] Weatherhead PJ , Sperry JH, Carfagno GLF, Blouin-Demers G (2012) Latitudinal variation in thermal ecology of North American ratsnakes and its implications for the effect of climate warming on snakes. J Therm Biol 37: 273–281. 10.1016/j.jtherbio.2011.03.008.

[ref96] Welbourne DJ , Paull DJ, Claridge AW, Ford F (2017) A frontier in the use of camera traps: surveying terrestrial squamate assemblages. Remote Sens Ecol Conserv 3: 133–145. 10.1002/rse2.57.

[ref97] Welsh AH , Cunningham RB, Donnelly CF, Lindenmayer DB (1996) Modelling the abundance of rare species: statistical models for counts with extra zeros. Ecol Model 88: 297–308. 10.1016/0304-3800(95)00113-1.

[ref98] Whitaker AH (1984) *Hoplodactylus kahutarae* n. sp. (Reptilia: Gekkonidae) from the Seaward Kaikoura Range, Marlborough, New Zealand. N Z J Zool 11: 259–270. 10.1080/03014223.1984.10428239.

[ref99] Wilson BS , Cooke DE (2004) Latitudinal variation in rates of overwinter mortality in the lizard *Uta stansburiana*. Ecology 85: 3406–3417. 10.1890/03-4075.

