# Supplementary material for: Extreme tolerance for nocturnal emergence at low body temperatures in a high-latitude lizard: implications for future climate warming
Source: Conserv Physiol. 2023 Jan 21;11(1):coac082. doi: 10.1093/conphys/coac082 (PMC9868685; doi:10.1093/conphys/coac082)
Supplement: Web_Material_coac082 [file web_material_coac082.zip › Supplementary info revision.docx]

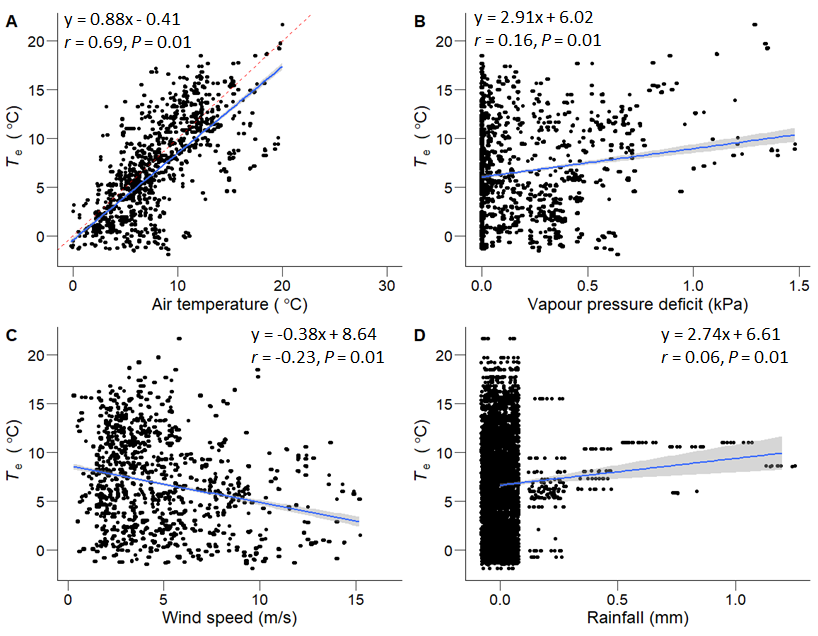


Figure S1: Plots of nighttime operative environmental temperature (*T_e_*) of copper models exposed on the rock surface for the first five hours after dusk at Macraes, Otago, New Zealand, as it relates to A). Air temperature. B). Vapour pressure deficit C). Wind speed and D). Rainfall. Data were pooled for all the seasons. The broken red line indicates the isothermal line.


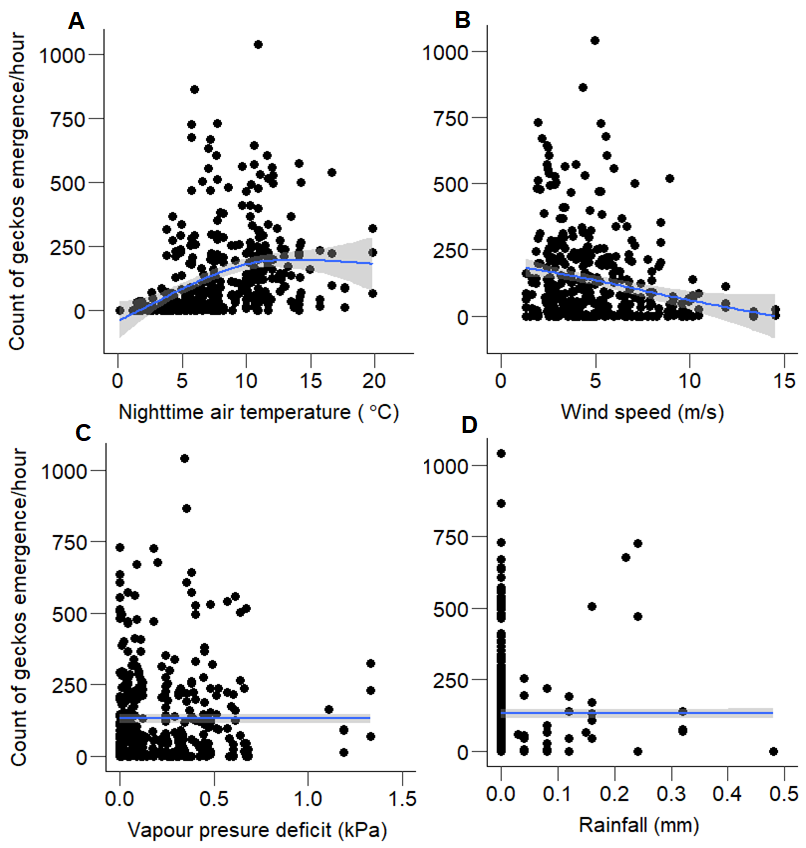


Figure S2: Counts of the gecko *Woodworthia* “Otago/Southland” sampled per hour vs. weather variables at Macraes, Otago, New Zealand. Air temperature, wind speed and rainfall data were obtained from a weather station 4km from the field study site. Data were pooled for all the seasons.


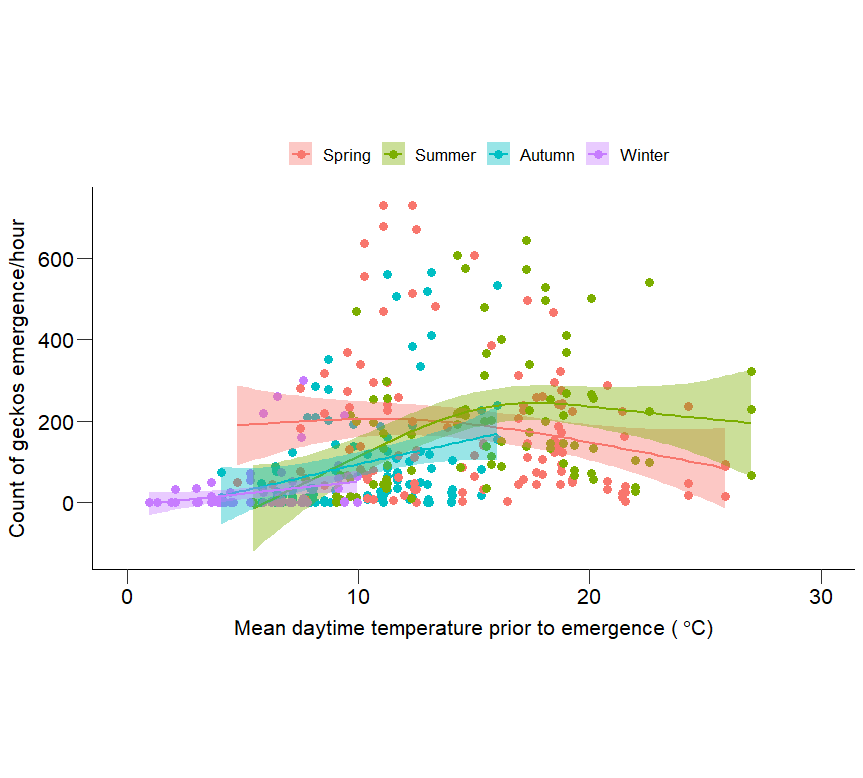


Figure S3: Relationship between nocturnal emergence of the gecko *Woodworthia* “Otago/Southland” per hour at Macraes, Otago, New Zealand and mean daytime air temperature before emergence. Nocturnal emergence is likely to be higher on nights with prior warmer temperatures, with more observed effects in winter and autumn, and to some extent in summer compared to spring. Outliers were removed after diagnostic plots.


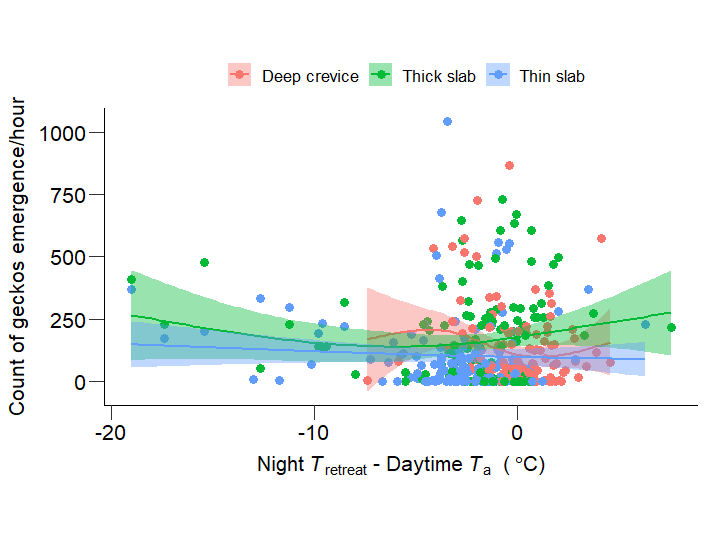


Figure S4: Relationship between nocturnal emergence of the gecko *Woodworthia* “Otago/Southland” per hour in different retreat types at Macraes, Otago, New Zealand, and the difference between retreat temperature at night and daytime air temperature. Nocturnal emergence activity was not significantly affected by the difference.
